# 3D Printed Anatomy-Specific Fixture for Consistent Glenoid Cavity Position in Shoulder Simulator

**DOI:** 10.1155/2018/2572730

**Published:** 2018-10-09

**Authors:** Gabriel Venne, Greg Esau, Ryan T. Bicknell, J. Tim Bryant

**Affiliations:** ^1^Department of Anatomy and Cell Biology, Faculty of Medicine, McGill University, QC, Canada; ^2^Department of Surgery, Kingston General Hospital, Queen's University, Kingston, ON, Canada; ^3^Department of Mechanical and Materials Engineering, Queen's University, Kingston, ON, Canada

## Abstract

**Purpose:**

Fixation methods for consistent anatomical structure positioning in biomechanical testing can be challenging. Image-based 3D printing is an attractive method for fabrication of biomechanical supports of anatomical structure due to its ability to precisely locate anatomical features with respect to the loading system.

**Method:**

A case study is presented to provide a design guide for fixation block fabrication. The anatomy of interest was CT scanned and reconstructed in 3D. The model was imported into commercially available CAD software and modified into a solid object and to create the fixture block. The CAD fixture block is standardized such that anatomical features are always in the same position for the testing system by subtracting the anatomy from a base fixture block.

**Results:**

This method allowed a strong immobilization of anatomical specimens and a controlled and consistent positioning feature with respect to the testing system. Furthermore, the fixture block can be easily modified and adapted to anatomical structures of interest using CAD software.

**Conclusion:**

This approach allows preservation of the bony anatomy integrity and provides a repeatable and consistent anatomical positioning with respect to the testing system. It can be adapted for other anatomical structures in various other biomechanical settings.

## 1. Background

The aging population is putting increased demands on health care. Age-related diseases like osteoarthritis are on the rise, underlining the need for increased research in various areas of orthopaedics, including joint biomechanics [1, 2]. This research field encompasses kinematic studies, mechanical testing of implants, and joint simulator development.

In a previous study, we found that additive-manufacturing, also known as fused deposition modeling or 3D printing, could produce bone models derived from CT imaging with an accuracy of less than half a millimeter [3]. This additive-manufacturing technology has also been highly useful in producing bony anatomical structures of complex shapes in preoperative planning [4–6]. It also allows the design and manufacture of complex shapes derived from the patient's anatomy, such as surgical drill-guides that fit the patient's bony anatomy surface for placing a prosthetic device with respect to the preoperative plan [7]. However, the use of these systems in surgical applications must also account for material properties such as strength and modulus [8, 9].

Anatomical support in biomechanical testing is intended to provide stabilization of the studied anatomy while external loads are applied. Immobilization and mounting techniques include potting with diverse materials, custom-designed fixtures [10], clamping, and direct fixturing using a variety of bearings and fasteners [11]. These methods, although robust, are challenging to apply and to ensure there is no loss of immobilization. Furthermore, the locations of specific anatomical features are challenging to align with respect to the fixture in 3D. As such, the use of custom-designed supports using additive manufacturing is attractive for the fabrication of biomechanical supports following the principles used in machine fixtures used in manufacturing applications. The purpose of this communication is to describe a standard procedure for 3D printing anatomy-specific support models in a variety of applications and to provide a design guide using a case study for a specific application to a complex fixture for the shoulder.

## 2. Overview

An anatomy-specific support is a structure in which the 3D-reconstructed anatomy of the region of interest has been subtracted to form a structural negative mold, termed a fixation block. The raw data of the specimen can be provided using medical imaging, 3D scanning, or other systems that can produce a 3D model. A reconstructed model is produced using image segmentation and processing software to isolate the anatomical region of interest. This model is then imported into computer-aided design (CAD) software and subtracted to form the support. Finally, the anatomy-specific support is printed in 3D.

### 2.1. Image Acquisition

The desired cadaveric anatomy was CT scanned at a high resolution using a 16-slice mobile gantry CT scanner (Lightspeed+XCR, General Electric, Milwaukee, WI, USA) using a slice thickness of 0.625 mm. The datasets were exported in Digital Imaging and Communications in Medicine (DICOM) file format.

### 2.2. 3D-CT Reconstruction

DICOM files were imported into commercial software (Mimics 15.0, Materialise, Leuven, Belgium) to segment the desired 2D-CT scan anatomy into a 3D-CT reconstructed model. A mask of the target anatomy was created using embedded bone thresholding settings. The mask was inspected in each of the three planes 2D-CT coordinates and edited to ensure accuracy of the anatomy representation. The 3D-CT reconstruction model was then constructed from the 2D-CT mask, and then exported as a stereolithography (∗.stl) file.

### 2.3. CAD of the Fixation Block

The 3D-CT reconstructed models in ∗.stl format were imported into commercially available software (SolidWorks™, Dassault Systèmes Waltham, MA). This permits the construction of a support by first defining an outer block and then subtracting the 3D-CT reconstructed model from the support.

### 2.4. Additive Manufacturing of the Fixation Block

The anatomic-specific support CAD model was digitally inspected and exported as a ∗.stl format to be 3D printed using additive manufacturing (Dimension SSt 1200es, Stratasys, Eden Prairie, USA). The anatomic-specific support was printed in Acrylonitrile Butadiene Sytrene (ABS) thermoplastic with a printer resolution of 0.254 mm. The model was finally cleaned of any printing-support material from the printer, inspected for any printing error and finally tested on the studied anatomy.

## 3. Case Study: Scapular Fixture for Shoulder Simulator

A system was previously designed to study the biomechanics of shoulder arthroplasty [12]. The scapula is fixed to a frame and cables used to represent the anterior, middle, and posterior heads of the deltoid. The cables are attached through ball joints to insertion points on the humerus and routed through pulleys to electric linear actuators. Inline load cells are used to measure force in each cable. Critical to this system is the location of muscle insertion and wrapping points determined from anatomical studies. As such, the use of an image-based reference frame enabled the positioning of the scapula with respect to the cable actuators that has distinct advantages compared to other fixation techniques. Specifically, the method reduces the effect that anatomical variations have on the positioning of deltoid and rotator cuff muscle origins relative to the centroid of the glenoid. By preserving the scapular anatomy, CAD models can be easily modified and reprinted, and the fixture can be removed without altering the anatomical structure.

### 3.1. Importing Files

Most scanners produce a surface, an infinitesimally thin sheet that covers the entire object. Before any CAD modifications can be done to a model, it must be converted into a solid file, such as a SolidWorks™ part file. This is not always straightforward, and SolidWorks™ has a *KNIT* tool that completes the surface and then attempts to fill it in, but this occasionally requires manual intervention.

### 3.2. Glenoid Cavity Centroid

**Figure 1:**
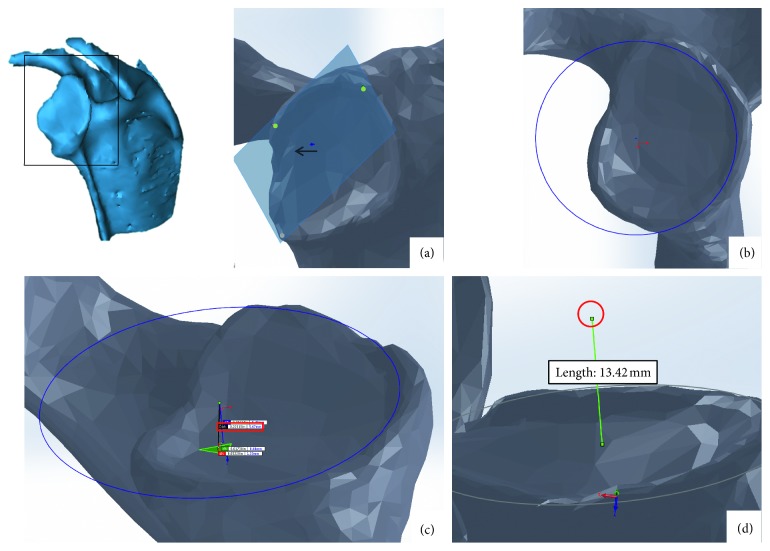
Glenoid cavity centroid: (a) the bearing surface is defined by a plane that rests on the three most distal vertices; (b) a circle is fitted to the defined bearing surface; (c) the normal distance is measured from the circle centroid to glenoid cavity surface; (d) the glenoid cavity centroid is then calculated.

When the∗.stl file has been successfully converted to a solid model file, the centroid of the glenoid cavity must be found to define reference planes (Figure 1). First, using CAD software, a face plane is defined that rests on the three most distal vertices of the glenoid cavity and must not intersect any part of the glenoid except those three points. The glenoid cavity is assumed to be a sphere, and its centroid is used in combination with the face plane for reference and measurement.

- There is a rim on the glenoid cavity that constrains superior motion of the shoulder. On the face plane, trace a circle that circumscribes the rim, with radius, *a*.
- Find the normal distance between the face plane and the glenoid cavity, *b*. In SolidWorks™, this can be done using the *MEASURE* tool. It is important to ensure the normal distance from the face plane is measured since the anatomical reference plane is not aligned with the *x*-*y*-*z* axes.
- The centroid of the glenoid is located on the normal of the face plane that intersects the centre of the circle. The distance along the normal from the plane is the radius of the traced circle (*a*) minus the normal distance (*b*).

### 3.3. Reference Planes

**Figure 2:**
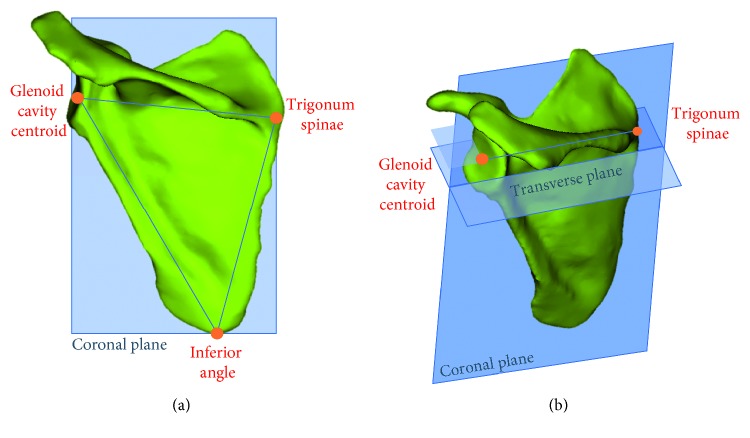
Reference planes: (a) coronal plane on scapula model; (b) mediolateral axis used in combination with the coronal plane to find the transverse plane and transverse and coronal plane are used to determine sagittal plane.

Although the reference planes are not anatomical planes, per se, they are used to relate the scapula to the biomechanical loading system [13] (Figure 2). These can be used to relate to the anatomical planes using digitized measurements, which is the main advantage of using an image-based fixation method.

- Coronal plane: the coronal plane consists of the glenoid cavity centroid, the trigonum spinae, and the inferior angle. The centroid of the trigonum spinae and the most inferior part of the inferior angle are selected for this definition.
- Transverse plane and mediolateral axis: the mediolateral axis is on the transverse plane and normal to the sagittal plane. The mediolateral axis consists of the line between the centroid of the trigonum spinae and glenoid cavity centroid. These must be the same points as those used to define the coronal plane. The transverse plane is thus defined as containing the mediolateral axis and as perpendicular to the coronal plane.
- Sagittal plane: The sagittal plane is perpendicular to the two other planes using the glenoid cavity centroid of the glenoid cavity as the origin.

### 3.4. Fixation Block Base

Fixation blocks are standardized such that the glenoid cavity centroid is always in the same position relative to the simulator. In general, a fixation block is made by extruding a rectangle of material from the transverse plane in the inferior direction. Guides are then extruded from the inferior surface to align the scapula in the ML and AP directions.

### 3.5. Parting Line

**Figure 3:**
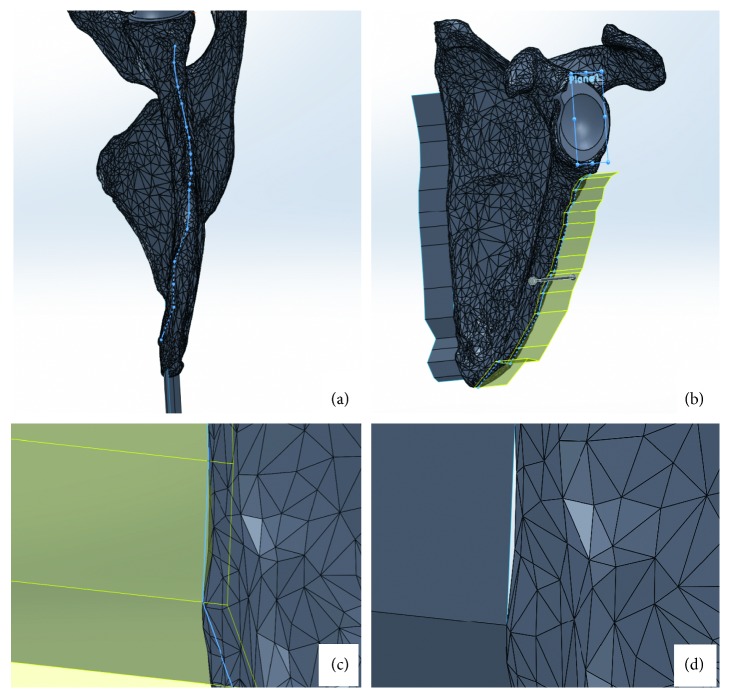
Parting line: (a) tracing parting line on lateral side of scapula; (b) extruding a plane from the traced parting line; (c) example of a gap between parting surface and scapula; (d) two-directional extrusion.

Once the CAD file for the fixation block has been made, it must be divided into sections so that it can be opened to insert the scapula (Figure 3). This division can be accomplished by creating planes that extrude mediolaterally from the scapula and then by cutting the fixation block with them.

Using two separate 3D sketches, the parting line is traced on the medial and lateral faces of the scapula. Surfaces are then extruded from the sketch. When extruding, the direction is specified from the reference planes. The extrusion should be normal to the sagittal plane. Because the 3D sketch may not consistently intersect the scapula, an extrusion in the opposite direction is specified to ensure that all gaps are closed. The surface must extend above and below the fixation block dimensions; it is much better to have a parting surface that is too large than too small.

### 3.6. Locating Fins

- The transverse profile of the block is sketched on the transverse plane and extruded downwards. It is important to treat the block as a separate body from the scapula. In SolidWorks™, this is done by unchecking *MERGE RESULT* in the *EXTRUSION FEATURE* window.
- Locating fins are extruded downwards from the inferior face of the block. Ensure these features *do* merge with the block.

### 3.7. Parting Features

**Figure 4:**
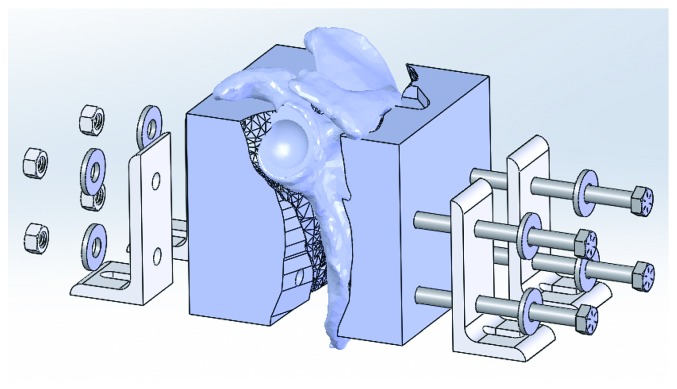
Final assembly with locking features shown. Note pocket in right segment.

- The scapula is subtracted from the fixation block, which leaves a cavity in the shape of the scapula (Figure 4). In SolidWorks™, this is done using the *COMBINE* command and selecting *SUBTRACT* rather than *ADD*. Subtracting the scapula from the block leaves the block and parting surfaces intact.
- Some CAD packages may be able to perform partial surface cuts; however, SolidWorks™ requires a workaround. This can be done by sketching a slot between the anterior and posterior midpoints with a very tiny radius (0.00001″). This slot is then used to create an extruded cut that is 0.00002″ thick through the entire model, forming two bodies. If prompted, ensure to keep both bodies.
- There are now two bodies in the CAD environment—both of which are halved by a parting plane. Use the surfaces to cut both bodies. SolidWorks™ is unable to cut with a surface and keep both sides, so this must be done for each side separately.
- When the fixation block has been cut with the surfaces, one half of the block will remain. It will be comprised of two bodies. Select both bodies and save them. SolidWorks™ gives the option of inserting the bodies into a part file in their original orientation.
- Before modifying these bodies, ensure that the links to the original model are broken. Select one side of the central cut, and create a sketch on it. Trace the outside perimeter of the face. In SolidWorks™, this can be done quickly by using the CONVERT ENTITIES command.
- This traced profile is extruded until it contacts the other body—this can be done by blindly extruding 0.00002″ or by specifying the adjacent surface as the limit. Ensure this extrusion merges the two entities.

### 3.8. Separation Clearance and Locking Features

For the fixation block to fit the scapula, it must only close on it in one direction (Figure 3). A pocket is used to perform this function by clearing material in the pull direction so that the feature that fits in the inadmissible pocket can be inserted and removed. In addition, features to lock the block to the testing system are added.

- When one side of the fixation block is completed, the direction of the surface cuts in the master model is flipped. This is repeated to create a single fixation block section. The fixation block sections may then be saved as∗.stl files and 3D printed. Typically, the blocks are printed at semisparse infill.
- Circular holes are added to align with the locking features of the testing system.

### 3.9. Application to Testing

The fixation blocks are on hand, and ready for testing by the time a cadaveric sample is removed from the freezer.

- Muscle tissue from the scapula is released to expose the bone and should fit snugly in the fixation block. However, 3D printing tends to produce extra material, so the cavity is undersized. A file, rasp, or sandpaper is used to make modifications to the fixation block parts until the scapula is firmly held. After pilot testing, this rectification step can be reduced if the fixation block is printed to be larger, typically by 2%.
- When the scapula is in the fixation blocks, holes are drilled in the scapula to align with the locking features incorporated in the design.

## 4. Summary and Conclusion

The purpose of this fixture block design method was to create anatomy-specific support for complex anatomical structures allowing a controlled placement and immobilization in a kinematic simulator. This method, using additive-manufacturing, allowed a strong immobilization of cadaveric scapulae and a controlled and consistent positioning of glenoid centroid with respect to the testing system. As well, the CAD of the fixture block can be easily modified and adapted to other anatomical structures of interest. No fixture block failure has been reported during our testing after multiple cycles of use.

The presented method was described for scapulae fixation for a shoulder simulator specifically. However, the same method has been very useful in our laboratory for various other biomechanical testing projects: distal radius and ulna fracture plate mechanical testing, foot prosthetic design and mechanical evaluation, and image-guided surgery platform design for *in vitro* testing [14, 15].

Additive manufacturing provides a good alternative for anatomy fixtures in any situation where biomechanical testing requires repeatable procedures to be applied on bony anatomy. Its integration with image-based solid modeling has advantages in its ability to locate anatomical landmarks precisely with respect to testing systems.

## Data Availability

No data were used to support this case study. All the necessary information that might be needed to reproduce the presented method is included within the article.

## References

[1] Johnson V. L., Hunter D. J. (2014). The epidemiology of osteoarthritis. *Best Practice & Research Clinical Rheumatology*.

[2] Neogi T., Zhang Y. (2013). Epidemiology of osteoarthritis. *Rheumatic Disease Clinics of North America*.

[3] Smith E. J., Anstey J. A, Venne G., Ellis R. E. (2013). Using additive manufacturing in accuracy evaluation of reconstructions from computed tomography. *Proceedings of the Institution of Mechanical Engineers, Part H: Journal of Engineering in Medicine*.

[4] Bizzotto N., Tami I., Santucci A. (2015). 3D Printed replica of articular fractures for surgical planning and patient consent: a two years multi-centric experience. *3D Printing in Medicine*.

[5] Dobbe J. G. G., Vroemen J. C., Strackee S. D., Streekstra G. J. (2013). Patient-tailored plate for bone fixation and accurate 3D positioning in corrective osteotomy. *Medical and Biological Engineering & Computing*.

[6] Sutradhar A., Park J., Carrau D. (2016). Designing patient-specific 3D printed craniofacial implants using a novel topology optimization method. *Medical and Biological Engineering & Computing*.

[7] Kunz M., Rudan J. F., Xenoyannis G. L., Ellis R. E. (2010). Computer-assisted hip resurfacing using individualized drill templates. *Journal of Arthroplasty*.

[8] Zanetti E. M., Bignardi C., Leondes C. T. Structural analysis of skeletal body elements: numerical and experimental methods. *Biomechanical Systems Technology*.

[9] Zanetti E. M., Aldieri A., Terzini M., Calì M., Franceschini G., Bignardi C. (2017). Additively manufactured custom load-bearing implantable devices: grounds for cautions. *Australasian Medical Journal*.

[10] Zanetti E. M., Bignardi C., Audenino A. L. (2012). Human pelvis loading rig for static and dynamic stress analysis. *Acta of Bioengineering and Biomechanics*.

[11] Zanetti E. M., Audenino A. L. (2010). Differential thermography for experimental, full-field stress analysis of hip arthroplasty. *Journal of Mechanics in Medicine and Biology*.

[12] Clouthier A. L., Hetzler M. A., Fedorak G., Bryant J. T., Deluzio K. J., Bicknell R. T. (2013). Factors affecting the stability of reverse shoulder arthroplasty: a biomechanical study. *Journal of Shoulder and Elbow Surgery*.

[13] Wu G., Van Der Helm F. C. T., Veeger H. E. J. (2005). ISB recommendation on definitions of joint coordinate systems of various joints for the reporting of human joint motion—part II: shoulder, elbow, wrist and hand. *Journal of Biomechanics*.

[14] Giles D. S., Kempston M. P., Sellens R. W. Locked versus non-locked plating for the fixation of radius fractures: a biomechanical comparison.

[15] Rasquinha B. J., Dickinson A. W. L., Venne G. (2015). Crossing-Lines Registration for Direct Electromagnetic Navigation. *Lect Notes Comput Sci*.

